# Smoking and Drinking

**Published:** 1886-07

**Authors:** 


**Smoking and Drinking.**—Tobacco blindness is becoming a common affliction. At present there are several persons under treatment for it at one London hospital. It first takes the form of color blindness, the sufferers who have smoked themselves into this condition being quite unable to tell the color of a piece of red cloth held up before them. Sometimes the victim loses his eyesight altogether. Although smoking is to a large extent the cause of the malady, and so gives it its naame, heavy drinking is also partly responsible.

**If a Summer Sick Room** has a fire-place put a candle in it. The upward draught makes an excellent system of ventilation, especially if a window be left open to allow fresh air ingress.

